# Coronavirus and the eye: what is relevant so far?

**DOI:** 10.5935/0004-2749.20200057

**Published:** 2020

**Authors:** Roberto Lauande, Jayter Silva Paula

**Affiliations:** 1 Departamento de Oftalmologia, União Metropolitana de Educação e Cultura, Lauro de Freitas, BA, Brazil; 2 Department of Ophthalmology, Otorrhinolaryngology and Head and Neck Surgery, Faculdade de Medicina de Ribeirão Preto, Universidade de São Paulo, Ribeirão Preto, SP, Brazil

COVID-19 is a respiratory disease caused by the enveloped, single-stranded RNA coronavirus, SARS-CoV-2, which has resulted in an increasing number of fatalities across the world. The first warnings on COVID-19, or SARS-CoV-2, were issued by Dr. Li Wenliang, an ophthalmologist in Wuhan province, China, in December 2019. Unfortunately, he died a little later, as a victim of the illness, at age 34 years. Emerging evidence suggests that Dr. Li could have been infected after contact with a patient who was asymptomatic at the time of consultation but presented the symptoms afterward^(1)^.

Recently, Wu et al.^(2)^ when examining a group of 38 hospitalized patients found signs of conjunctivitis in almost 32% of cases to be more pronounced in the sickest ones. Those patients were admitted due to their more severe signs and symptoms, with a probable diagnosis of COVID-19 [28 of them with positive viral RNA confirmation in the oropharynx secretion, tested using polymerase chain reaction with reverse transcription (RT-PCR)]. Histological signs of the viral infection were detected in two conjunctival samples of the 11 patients tested (18%). These results have been considered as the first confirmation of the viral invasion to the conjunctiva and were sufficient to emphasize the vital advice of protecting health professionals who would be dealing with tears and ocular secretions of patients with suspected COVID-19 and red eyes. In addition, these authors emphasized that conjunctivitis may be a relatively common finding in patients with COVID-19-related pneumonia. The most common clinical picture of patients with conjunctivitis observed in this case series included hyperemia, tearing, chemosis, and mucous discharge.

Subsequent reports suggested a typical follicular conjunctivitis in patients with COVID-19. Chen et al.^(3)^ reported that 25 of 534 patients with COVID-19 (4.7%) showed conjunctival congestion, and in three of them, that was the very initial clinical sign described. Conjunctival congestion had persisted for approximately 5 days, with a range of 2-10 days, associated with dry eye symptoms (21%), blurred vision (13%), and foreign body sensation (12%). The majority of patients had eye complaints associated with fever and respiratory symptoms, including dry cough and shortness of breath.

Therefore, this situation arises a crucial question, i.e., is it possible that tears of patients with confirmed COVID-19, without signs of conjunctivitis, can infect health professionals?

Notwithstanding all the strongly recommended care procedures when dealing with any secretions of patients with COVID-19, and the potential presence of viral RNA in the conjunctiva, a few studies have suggested a relatively low probability of detection of virus in the tears of patients with COVID-19 who do not manifest the clinical signs of conjunctivitis.

In a small study, no viral RNA was detected in either tears or conjunctival secretions of 30 patients with SARS-CoV-2 who presented moderate or severe respiratory symptoms and no signs of conjunctivitis^(4)^. These patients had detectable RNA levels in the oropharynx samples, and only one patient with conjunctivitis and severe symptoms of COVID-19 had viral RNA in tears.

In a later study, consisting of 72 patients with SARS-CoV-2 confirmed by RT-PCR at the Tongji School of Medicine, only two patients had conjunctivitis (2.8%)^(5)^. Only one of them, with signs of conjunctivitis, had viral RNA detected in tears. None of the other 70 patients, without signs of conjunctivitis, had their tears tested positive for COVID-19.

Considering the unaccountable frequency of conjunctival congestion and confirmation of the presence of COVID-19 in the eyes, we may discuss safety for the use of contact lenses. For the health professionals, both eye protection (goggles or face shield) and other appropriate personal protective equipment (PPE) are mandatory in a hospital environment, irrespective of the use of contact lenses or corrective glasses. The general public has been recommended to wear glasses instead of contact lenses, as glasses may be an additional physical barrier to the aerosol particles that can potentially reach the eyes.

It is important to consider the potential risk of contamination due to the handling of contact lenses, including the well-known fact that patients who wear glasses tend to scratch and manipulate the eyes less compared with contact lens wearers. Although speculative, the contact lens surfaces, mostly manufactured using polymethyl acrylate and/or silicone and their derivatives, as well as the lens storage boxes, can harbor the virus for several hours and even days. We believe that wearing glasses is the safest general option during this COVID-19 outbreak. However, if one chooses to continue using contact lenses, extreme care must be taken regarding hand washing when handling the lenses, in addition to not scratching the eyes or face, and to the use of PPE when attending a hospital setting.

In the actual context, what could be the recommendations for health professionals who are caring for patients with conjunctivitis and suspected COVID-19? The recommended procedures, when caring for patients with suspected or confirmed COVID-19, comprise the protection of the mouth, nose, and eyes using surgical masks (or N-95 masks in hotspots) and either goggles or face shields. Furthermore, breath shields attached to slit-lamps are useful to protect ophthalmologists^(6)^.

To prevent the transmission of COVID-19, the same disinfection practices that are already used to prevent the spread of other viral pathogens commonly found in clinical practice should be reinforced. Finally, in addition to the guidance of rescheduling elective visits and surgeries, cleaning and disinfection must be performed before and after examining each patient, because SARS-CoV-2 has been detected up to few days in some surface materials (see https://www.cdc.gov/coronavirus/2019-ncov/prevent-getting-sick/cleaning-disinfection.html?CDC_AA_refVal=https%3A%2F%2Fwww.cdc.gov%2Fcoronavirus%2F2019-ncov%2Fprepare%2Fcleaning-disinfection.html).
